# Mucinous Signet-Cell Adenocarcinoma of the Ileum: A Diagnostic Challenge—Case Report and Review of the Literature

**DOI:** 10.1155/2022/5703407

**Published:** 2022-05-28

**Authors:** Tiago Martins, Jalaluddin Umar, Kevin Groudan, Hariharan S. Bharadwaj, David Desilets

**Affiliations:** ^1^Baystate Medical Center, Internal Medicine Residency, 759 Chestnut Street, Springfield, MA 01199, USA; ^2^Baystate Medical Center, Gastroenterology Department, 759 Chestnut Street, Springfield, MA 01199, USA; ^3^Baystate Medical Center, Pathology Department, 759 Chestnut Street, Springfield, MA 01199, USA

## Abstract

Malignancies of the small intestine are rare. Signet-ring cell carcinoma (SRCC) is one of the rarest forms of adenocarcinoma that can arise in the small intestines. We present a case of a patient who originally presented with abdominal pain and radiographic findings suggestive of ileal congestion. The ileal biopsy specimens were nonspecific, and the patient began a trial of corticosteroid treatment for suspected Crohn's disease. A repeat colonoscopy yielded biopsies that were positive for malignancy. The patient then underwent an exploratory laparotomy which led to the diagnosis of SRCC. Given their similar presentations and the extreme rarity of this unusual malignancy, it can be difficult to differentiate between new-onset Crohn's disease and SRCC. A review of the literature was conducted to provide us with an improved understanding of previously documented cases of SRCC.

## 1. Introduction

Primary malignancies are detected only rarely in the small intestine. Small intestinal lesions represent only ∼2.3% of all malignancies identified in the digestive system and ∼0.42% of malignancies overall [1]. Signet-ring cell carcinoma (SRCC) is a rare subtype of adenocarcinoma that is identified most frequently in the stomach, where it accounts for 25% of all gastric cancers. SRCC has also been detected in the pancreas, breasts, bladder, ovaries, esophagus, lungs, and large intestine [2, 3]. The five-year survival rate of patients diagnosed with SRCC has been estimated at 20–30% [1]. While patients diagnosed with Crohn's disease (CD) have an increased risk of developing adenocarcinoma of the ileum compared to the general population, [4] the precise mechanisms underlying this observation remains unknown [4–7]. In this report, we present the case of a patient who was initially treated for presumed new-onset CD before receiving a correct diagnosis of SRCC, and so, we review how both diagnoses can present similarly and pose a diagnostic challenge.

## 2. Case

The patient was a 68-year-old woman with a prior hysterectomy and family history of CD who presented as a transfer from an outside hospital for the evaluation and treatment of recurrent severe lower abdominal pain.

The patient had initially presented to the outside hospital four weeks prior to this transfer with a chief complaint of constant lower abdominal pain without diarrhea, but with intermittent constipation. The patient also reported a four-pound weight loss. The patient's social history was negative for smoking, alcohol, or illicit drug use. She reported that her mother had been diagnosed with CD. An initial computed tomography (CT) scan revealed abnormal wall thickening and fat stranding within the terminal ileum consistent with an infectious or inflammatory process with no visible abscesses. The mesenteric lymph nodes in the right lower quadrant were also mildly prominent consistent with mesenteric adenitis, and neoplastic disease could not be ruled out. A follow-up colonoscopy revealed a redundant colon with normal mucosa except for superficial, linear erosions in the distal rectum, sigmoid diverticulosis, and an 8 mm polyp in the ascending colon. The terminal ileum appeared to be diffusely congested without any ulcerations or obvious inflammation.

No evidence of pathology was noted on biopsy specimens obtained from the ileocecal valve. Laboratory studies from the outside hospital included a complete blood count (CBC) that revealed slight anemia (hemoglobin at 12 g/L and hematocrit at 37.5%) with normal white blood cell (WBC; 10.2 cells/mcL) and platelet counts (338 cells/mcL). Her routine serum chemistry panel was also within normal limits. The patient had a negative T-spot for tuberculosis and a negative hepatitis panel; elevated levels of C-reactive protein (CRP; 45 mg/L) and calprotectin (408 *μ*g/mL) levels were noted. Based on the family history, colonoscopy findings, and elevated levels of CRP and calprotectin, the patient was initially diagnosed with CD and began treatment with 60 mg methylprednisolone. Her symptoms initially improved on this regimen. She transitioned to oral prednisone and was discharged four days later.

Ten days after discharge, the patient returned to the same outside hospital with a recurrence of persistent abdominal pain. Results from a second CT scan were similar to those reported previously, including the possibility of an infectious process. The patient was hospitalized for an additional four days and was discharged with a treatment plan that included ciprofloxacin and metronidazole together with prednisone as previously prescribed. She was scheduled to complete a prednisone taper and begin treatment with 5 mg/kg infliximab on an outpatient basis.

Two days after the second hospital discharge, the patient returned with worsening abdominal pain. A third abdominal CT performed at this time revealed terminal ileal wall thickening with surrounding fat stranding. Free fluid was detected, and ileus was also documented. The CT report noted that a portion of the inflamed segment may have developed an abscess or phlegmon.

**Figure 1:**
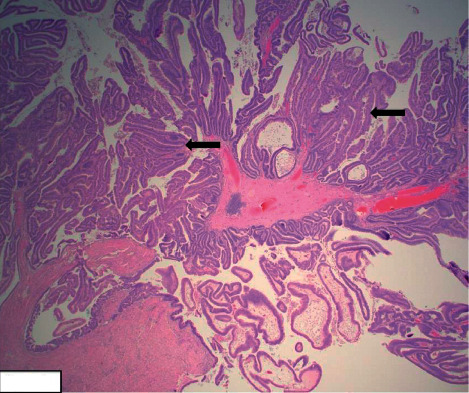
Tubulovillous adenoma with high-grade dysplasia. Ileal polyp showing slender, fingerlike projections formed by fibrovascular cores and lined by high-grade dysplastic epithelium (arrows) (H&E stain, 2×).

Given the ongoing complexity of this case, the patient was transferred to our tertiary care center. Upon arrival, she was hemodynamically stable (temperature, 36.7°C; pulse, 65 beats/min; blood pressure, 166/66 mmHg; SpO_2_, 99% on room air; and respiratory rate, 21 breaths/min). Her CBC documented slight anemia with a normal WBC count and normal serum chemistries. Liver function tests, including serum lipase, were all unremarkable. The patient continued to experience abdominal pain without nausea or diarrhea. She denied any overt gastrointestinal bleeding, fever, or chills. Her initial diagnosis was an exacerbation of CD with ileus and a potential abscess/phlegmon. The gastroenterology team performed a repeat colonoscopy, and a biopsy of the terminal ileum was obtained; the specimen revealed mild inflammatory changes and congestion. Pathological analysis of this specimen revealed a malignant process. Microscopic evaluation revealed tubulovillous adenoma with high-grade dysplasia in Figure 1. This was then confirmed with immunohistochemistry. The cells were negative for cytokeratin 7 but positive for cytokeratin 20 and CDX 2. Although signet-ring cell carcinoma of the small bowel can occur, its relative rarity combined with lack of in situ tumor favored metastatic disease. However, biopsy specimens collected from a subsequent esophagogastroduodenoscopy were grossly negative for malignancy.

The patient underwent an exploratory laparotomy which resulted in the resection of the ileocolic mass; a right-sided colectomy, ileostomy, and abdominal washout were performed, and a mucous fistula was created. The mesentery and the mass adhered to the intestinal wall. An abscess with pus drainage and significant induration were also noted, which suggested an inflammatory process and/or lymphadenopathy. The tumor was 25 cm in length and poorly differentiated and was invading the visceral peritoneum and appendix. With this final resection, the pathology team confirmed the final diagnosis but was also able to find in situ components. The tumor was noted to be arising in a background of tubulovillous adenoma of the terminal ileum, with high-grade dysplasia. Based on the characteristics of this malignancy, the patient was deemed to be a good candidate for adjuvant chemotherapy. A FOLFOX (folinic acid, fluorouracil, and oxaliplatin) regimen was initiated. She is currently on her third cycle of chemotherapy and doing well. This treatment regimen aims to eliminate the disease, reduce the risk of its recurrence, and improve overall survival.

## 3. Discussion

The case presented in this report illuminates the diagnostic challenge presented by SRCC in the ileum. Of note, this diagnosis was reached only after multiple hospitalizations and ultimately required a multidisciplinary approach. SRCC is considered to be a diffuse type of malignancy with limited cell cohesion. This property permits the malignant cells to infiltrate the visceral wall and precludes the formation of a visible mass [8]. Primary gastric SRCC can spread to the colon through lymphatic vessels and can target the transverse colon via the gastrocolic ligament [9]. While a few case studies that describe SRCC in the ileum have already been published, this case has several unique features, including multiple admissions and specific diagnostic tests that were performed before reaching the correct diagnosis.

SRCC in the colon or small intestines may mimic the signs and symptoms of CD [10]. SRCC presents with many features that are similar to those of CD, including long segments of concentric small intestinal thickening, obstructions, and ulcerations as well as intraluminal masses [5, 10, 11]. Patients with SRCC may also experience nausea, vomiting, abdominal pain, fistulae, hemorrhage, and perforation [12–14]. Many of these symptoms overlap with those of CD, and it can be difficult to distinguish between CD and small intestinal malignancy based on these findings alone [14–16]. Nevertheless, malignancy should certainly emerge as more prominent in the differential diagnosis if these symptoms prove to be refractory to standard CD therapy and bowel obstruction becomes evident [13]. Finally, it is important to note that patients diagnosed with CD are most likely to develop colorectal malignancies at 45–50 years of age [12, 13]. By contrast, de novo small intestinal malignancies typically develop in patients within the 60–69 year age bracket [15]. The major risk factor for CD presented by this patient other than her symptoms was her positive family history, which has been reported to increase the risk of CD by 3.22-fold [16].

**Figure 2:**
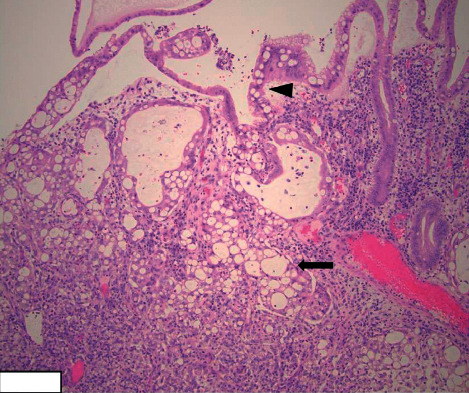
Invasive signet-ring cell carcinoma of the ileum (arrow) arising from dysplastic epithelium (arrowhead) (H&E stain, 20×).

**Figure 3:**
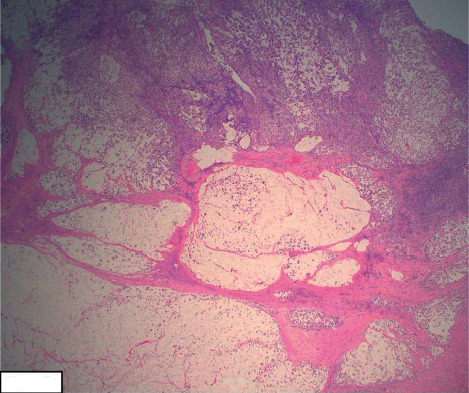
Invasive signet-ring cell carcinoma of the ileum with dissecting extracellular mucin pools. The tumor is seen invading the muscularis propria (H&E stain, 2×).

**Figure 4:**
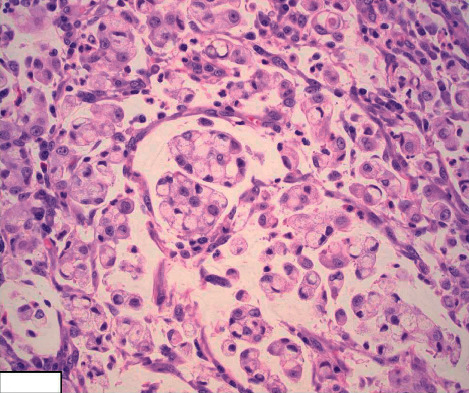
Image showing the signet-ring cell morphology at higher magnifications (H&E stain, 40×).

Given that the presenting signs and symptoms of SRCC can mimic those of CD, the most important diagnostic test leading to an accurate diagnosis is a colonoscopy and biopsy. The characteristic findings of SRCC include signet-ring cells, as shown in Figures 2–4. Histologically, SRCC cells produce mucin and have mucin vacuoles that displace the nucleus to the periphery of the cell [17]. It is important to note that approximately 50% of cases of poorly differentiated small intestinal malignancies that produce mucin, as in the case presented here, have a poor prognosis [4]. The current treatment options for small intestinal malignancies include wide resection that includes the mesentery and corresponding lymph nodes. The use of adjuvant chemotherapy has been described only in small retrospective studies [5].

This case report is important because it highlights the importance of considering malignancy in the differential diagnosis in cases of newly-diagnosed inflammatory bowel disease that do not respond to treatment. Despite its rarity, clinicians should maintain suspicion of SRCC even in the absence of immediate histological confirmation.

## Data Availability

No data were used to support this study.

## References

[1] Jemal A., Siegel R., Ward E., Hao Y., Xu J., Thun M. J. (2009). Cancer statistics, 2009. *CA: A Cancer Journal for Clinicians*.

[2] Dogan S., Celikbilek M., Eroglu E. (2013). Signet ring cell carcinoma mimicking ileal Crohn’s disease. *Gastroenterology Insights*.

[3] Paparo F., Piccardo A., Clavarezza M. (2013). Computed tomography enterography and 18F-FDG PET/CT features of primary signet ring cell carcinoma of the small bowel in a patient with Crohn’s disease. *Clinical Imaging*.

[4] Valério F., Cutait R., Sipahi A., Damião A., Leite K. (2006). Cancer in Crohn’s disease: case report. *Revista Brasileira de Coloproctologia*.

[5] Cahill C., Gordon P. H., Petrucci A., Boutros M. (2014). Small bowel adenocarcinoma and Crohn’s disease: any further ahead than 50 years ago?. *World Journal of Gastroenterology*.

[6] Jess T., Winther K. V., Munkholm P., Langholz E., Binder V. (2004). Intestinal and extra-intestinal cancer in Crohn’s disease: follow-up of a population-based cohort in Copenhagen County, Denmark. *Alimentary Pharmacology & Therapeutics*.

[7] Ramos C. R., Guillen P., Palomo M. J. (1997). Adenocarcinoma de intestine Delgado y enfermedad de Crohn. *Revista Española de Enfermedades Digestivas*.

[8] Hommel C., Knoedler M., Bojarski C. (2012). Diffuse gastric cancer with peritoneal carcinomatosis can mimic crohn’s disease. *Case Reports in Gastroenterology*.

[9] Katon R. M., Brendler S. J., Ireland K. (1989). Gastric linitis plastica with metastases to the colon. *Journal of Clinical Gastroenterology*.

[10] Winter M. W., Dokmak A., Marnoy Z., Sinagare S., Levy A. N. (2018). Metastatic gastric signet ring cell carcinoma mimicking Crohnʼs disease. *ACG Case Reports Journal*.

[11] Zenda T., Taniguchi K., Hashimoto T. (2007). Metastatic colon cancer mimicking Crohn’s disease. *Annals of Diagnostic Pathology*.

[12] Dossett L. A., White L. M., Welch D. C. (2007). Small bowel adenocarcinoma complicating Crohn’s disease: case series and review of the literature. *The American Surgeon*.

[13] Widmar M., Greenstein A. J., Sachar D. B., Harpaz N., Bauer J. J., Greenstein A. J. (2011). Small bowel adenocarcinoma in Crohn’s disease. *Journal of Gastrointestinal Surgery*.

[14] Greenstein A. J. (2000). Cancer in inflammatory bowel disease. *The Mount Sinai journal of medicine, New York*.

[15] Negri E., Bosetti C., La Vecchia C., Fioretti F., Conti E., Franceschi S. (1999). Risk factors for adenocarcinoma of the small intestine. *International Journal of Cancer*.

[16] Shen B., Remzi F. H., Hammel J. P. (2009). Family history of Crohnʼs disease is associated with an increased risk for Crohnʼs disease of the pouch. *Inflammatory Bowel Diseases*.

[17] Elsevier (2007). *Elsevier’s Integrated Pathology, by Thomas Charles King Mosby Elsevier*.

